# Supplementary material for: Short-term power prediction of photovoltaic power stations based on Kepler optimization algorithm and VMD-CNN-LSTM model
Source: PLoS One. 2025 Sep 25;20(9):e0329821. doi: 10.1371/journal.pone.0329821 (PMC12463232; doi:10.1371/journal.pone.0329821)
Supplement: Attachment — Submitted filename: Response to Reviewers.docx [file pone.0329821.s003.docx]

**Original Manuscript ID:** PONE-D-25-26679

**Original Article Title: “Short-term power prediction of photovoltaic power stations based on Kepler algorithm and VMD-CNN-LSTM model** ”

**To:** PLOS ONE Editor

**Re:** Response to reviewers

Dear Editor and reviewers,

On behalf of all the contributing authors , I would like to express our sincere appreciation of your letter and reviewers ' constructive comments concerning our entitled “Short-term power prediction of photovoltaic power stations based on Kepler algorithm and VMD-CNN-LSTM model” (Original Manuscript ID: PONE-D-25-26679).I have considered the comments very carefully and have revised the paper accordingly . These comments are all valuable and helpful for improving our article . We have extensively modified our manuscript according to the editor and reviewers ' comments We hope that the corrections are satisfactory . The reviewer's comments are listed below in green, with specific questions numbered. Our responses are presented in black, and changes or additions to the manuscript are marked in red. Point - by - point answers to the kind editor and two kind reviewers are listed below this letter .This study was supported by the Basic Research Fund for Higher Education Institutions in Hebei Province (Funding number: 2024QNJS09). In fact, this research originated from a project obtained by the corresponding author of the paper (Project number: 2024QNJS09), which is also funded by the Basic Research Fund for Higher Education Institutions in Hebei Province.We are uploading (a) a rebuttal letter that responds to each point raised by the academic editor and reviewer(s). (b)a marked-up copy of your manuscript that highlights changes made to the original version. and (c) an unmarked version of your revised paper without tracked changes.

Thanks again for your consideration of publishing our manuscript in your journal . We are looking forward to hearing from you soon .

Best regards,

<Jiangli Yu, Gaoyi Liang, Lei Wang*, Huiyuan He,Yuxin Liu,Qi Liu,Xinjie Cui and Hao Wang> et al.

杂志

**Reviewer#1, Concern # 1 :** The title can be modified to avoid using abbreviations, which can be more effective academically.

**Author response:**Thank you very much for taking time out of your busy schedule to review our article. We are sorry that we have some mistakes. We have modified the title according to your opinion and changed it to Short-term power prediction of photovoltaic power stations based on Kepler optimization algorithm and VMD-CNN-LSTM model. Please review it.

**Author action:**

**(title)**

**Short-term power prediction of photovoltaic power stations based on Kepler optimization algorithm and VMD-CNN-LSTM model**

**Reviewer#1, Concern # 2 :** If you use abbreviation, it's often helpful to spell it out in the abstract to clarify meaning for readers and reviewers. Some abbreviations are not declared, such as VMD-CNN-LSTM and KTH. As reviewer I understand sometimes if a common abbreviation in the field no need to be declared, but assume a reader is reading this without pervious knowledge or background.

**Author response:** Thank you very much for taking the time to review our article amidst your busy schedule. We apologize for any errors we may have made. We have added introductions to VMD, CNN, and LSTM in the abstract based on your feedback, and revised the abbreviation KTH. This will help readers understand even if they lack prior knowledge. Please review it.

**Author action:**

**(Paragraph 4 of the summary)**

It is a model combining variational mode decomposition (VMD), convolutional neural network (CNN) and long short term memory network (LSTM)

**(2.2, line 12)**

Among them, u_k(t)represents the 𝑘th IMF component, ω_k is center frequency,H(⋅) indicates the Hilbert transform,α is the parameter used to control the bandwidth penalty, and T is the length of the time window.

**Reviewer#1, Concern # 3 :** The abbreviation needs to be clear such as Kepler Optimization Algorithm (KOA) and not misleading the reader.

**Author response:** Thank you very much for taking time out of your busy schedule to review our article. We are sorry that we have made some mistakes. We have modified the abbreviation of Kepler optimization algorithm in the abstract according to your opinion, so that readers can understand it even if they do not have relevant knowledge. Please review it.

**Author action:**

**(Paragraph 7 of the summary)**

**Meanwhile, the Kepler optimization algorithm (KOA) is deeply integrated with this model to optimize the entire process of the model from data preprocessing to result correction.**

**Reviewer#1, Concern # 4 :** Abbreviations need to be declared in the first time mentioned in the text, as an example Long Short-Term Memory (LSTM) is declared in section III, while it has been mentioned before in different sections.

**Author response:** Thank you very much for taking time out of your busy schedule to review our article. We are sorry that we made some mistakes. We have added the introduction of LSTM (Long Short Term Memory Network) at the first place where it appears in the text according to your opinion, so that readers can understand clearly. Please review it.

**Author action:**

**(Paragraph 7 of the summary)**

**Meanwhile, the Kepler optimization algorithm (KOA) is deeply integrated with this model to optimize the entire process of the model from data preprocessing to result correction.**

**Reviewer#1, Concern # 5 :** In section 4.1, the word chapter has been highlighted. It is better to replace with word section to keep the academic consistency.

**Author response:**Thank you very much for taking time out of your busy schedule to review our article. We are sorry that we made some mistakes. We have changed the word chapter in section 4.1 to section according to your suggestion to maintain academic consistency. Please review it.

**Author action:**

**(4.1, first line)**

**The content of this section is to conduct experiments on KOA. KOA is selected as the experimental group, and the SSA algorithm, PSO algorithm, GWO algorithm, and WOA algorithm are selected as the control group.**

**Reviewer#1, Concern # 6 :** The performance of the model relies heavily on the quality and quantity of historical data. Poor or missing historical data can lead to poor predictions. The paper does not explore how varying levels of data quality can influence model performance.

**Author response:** Thank you very much for taking the time to review our article amidst your busy schedule. We apologize for any errors we may have made. Based on your feedback, we have added a dataset of 130MW (Station Site 2) in Section 5 and conducted a comparison of experimental tables and images to explore how varying levels of data quality affect model performance. The VMD-KOA-CNN-LSTM model outperforms other models as shown in the tables and images. Please review it.

**Author action:**

**V. Case verification and result analysis**

| Model name | MAE | RMSE | R² |
| --- | --- | --- | --- |
| CNN-LSTM | 5.18 | 6.81 | 0.93 |
| VMD-LSTM | 2.33 | 3.27 | 0.98 |
| KOA-CNN-LSTM | 4.53 | 6.10 | 0.98 |
| VMD - CNN - LSTM（Notoptimized） | 2.57 | 3.43 | 0.94 |
| VMD-KOA- CNN - LSTM | 2.26 | 3.13 | 0.98 |

**Reviewer#1, Concern # 7 :** The performance of the model is heavily reliant on the quality and quantity of historical data. If the historical data is not comprehensive or contains factual inaccuracies, then it could lead to worse predictions. The paper does not discuss how variations in the quality of data may impact the performance of the model.

**Author response:** Thank you very much for taking the time to review our article amidst your busy schedule. We apologize for any errors we may have made. Based on your feedback, we have added a dataset of 130MW (Station Site 2) in Section 5 and conducted a comparison of experimental tables and images to explore how changes in data quality affect model performance. The tables and images show that the VMD-KOA-CNN-LSTM model outperforms other models. Please review these additions.

**Author action:**

**V. Case verification and result analysis**

| Model name | MAE | RMSE | R² |
| --- | --- | --- | --- |
| CNN-LSTM | 5.18 | 6.81 | 0.93 |
| VMD-LSTM | 2.33 | 3.27 | 0.98 |
| KOA-CNN-LSTM | 4.53 | 6.10 | 0.98 |
| VMD - CNN - LSTM（Notoptimized） | 2.57 | 3.43 | 0.94 |
| VMD-KOA- CNN - LSTM | 2.26 | 3.13 | 0.98 |

**Reviewer#1, Concern # 8 :** The model's performance is highly reliant on the quantity and quality of historical data. If historical data lacks completeness or accuracy, it could lead to poor predictions. How variations in data quality can impact the model's performance is not covered in the research.

**Author response:** Thank you very much for taking the time to review our article amidst your busy schedule. We apologize for any errors we may have made. Based on your feedback, we have added a dataset of 130MW (Station Site 2) in Section 5 and conducted a comparison of experimental tables and images to explore how changes in data quality affect model performance. The VMD-KOA-CNN-LSTM model outperforms other models as shown in the tables and images. Please review it.

**Author action:**

**V. Case verification and result analysis**

| Model name | MAE | RMSE | R² |
| --- | --- | --- | --- |
| CNN-LSTM | 5.18 | 6.81 | 0.93 |
| VMD-LSTM | 2.33 | 3.27 | 0.98 |
| KOA-CNN-LSTM | 4.53 | 6.10 | 0.98 |
| VMD - CNN - LSTM（Notoptimized） | 2.57 | 3.43 | 0.94 |
| VMD-KOA- CNN - LSTM | 2.26 | 3.13 | 0.98 |

**Reviewer#1, Concern # 9 :** The performance of the model is highly dependent on the quantity and quality of historical data. If historical data is not complete or is inaccurate, it may result in suboptimal

**Author response:** Thank you very much for taking the time to review our article amidst your busy schedule. We apologize for any errors we may have made. Based on your feedback, we have added a dataset of 130MW (Station Site 2) in Section 5 and conducted a comparison of experimental tables and images to explore how changes in data quality affect model performance. The VMD-KOA-CNN-LSTM model outperforms the other models, as shown in the tables and images. Please review it.

**Author action:**

**V. Case verification and result analysis**

| Model name | MAE | RMSE | R² |
| --- | --- | --- | --- |
| CNN-LSTM | 5.18 | 6.81 | 0.93 |
| VMD-LSTM | 2.33 | 3.27 | 0.98 |
| KOA-CNN-LSTM | 4.53 | 6.10 | 0.98 |
| VMD - CNN - LSTM（Notoptimized） | 2.57 | 3.43 | 0.94 |
| VMD-KOA- CNN - LSTM | 2.26 | 3.13 | 0.98 |

**Reviewer#1, Concern # 10 :** In addition, for more insight to the work. These key points need to clear to give strength weight academically. How does Kepler Algorithm enhance prediction accuracy? What data sources were used for historical analysis? How does VMD impact data complexity reduction?

**Author response:** Thank you very much for taking the time to review our article amidst your busy schedule. We apologize for any errors we may have made. We have added to Section 5.3, based on your feedback, the significance and algorithmic advantages of optimizing the hyperparameters of the CNN-LSTM in KOA, explaining how the Kepler algorithm enhances prediction accuracy. We also discussed the complexity of photovoltaic data and the necessity of VMD decomposition, detailing how VMD technology reduces data complexity. In Section 5, we have included two datasets: one from the State Grid Renewable Energy Power Generation Prediction Competition in China, which includes data from two 50MW and 130MW photovoltaic power stations (Station site1 and Station site2). Please review these additions.

**Author action:**

**(V. Case verification and result analysis)**

**This study utilized data from two 50MW and 130MW photovoltaic power stations (Station site1 and Station site2) in China's State Grid Renewable Energy Power Generation Forecasting Competition [17].**

Figure 16 and 20 present the comparison between the prediction results of the KOA-CNN-LSTM and VMD-KOA-CNN-LSTM models and the actual power curves. It is evident that while the KOA-CNN-LSTM model can capture some temporal variations, it exhibits lag and insufficient accuracy when dealing with complex meteorological conditions and long-term trends. Photovoltaic power generation is highly non-stationary and complex, with fluctuations resulting from the superposition of multiple physical processes at various time scales: from cloud shadow shifts and equipment noise at the second level, to local meteorological changes and cloud evolution over minutes to hours, to weather system activities on a daily to weekly scale, and even diurnal and seasonal changes. These fluctuations at different frequencies are intertwined in the original power signal, making it challenging for direct prediction models to accurately capture their underlying patterns and are sensitive to noise.

To address the aforementioned challenges, Variational Mode Decomposition (VMD) can adaptively decompose complex signals into a series of physically meaningful eigenmode functions (IMFs) based on the frequency characteristics of the signal. Each IMF has a well-defined central frequency, clearly corresponding to core driving factors at different time scales, such as cloud shadows, air mass movements, and day-night or seasonal cycles. This decomposition achieves signal decoupling:

(1) Eliminate interference: can identify and filter out high frequency noise (IMF1-2).

(2) Separation of prediction focus: the predictable components at different scales (short-term, medium-term and long-term) (such as local cloud change (IMF3), weather system influence (IMF4), day and night patterns and seasonal trends (IMF5-6)) are effectively separated to avoid mixing.

(3) Reduce data complexity and enhance modeling accuracy: The decomposition of sequences enhances their stability and regularity, making it possible to tailor prediction models for different frequency components (for example, using short-term response models for high-frequency components and trend capture models for low-frequency components). This approach ultimately leads to more accurate overall power predictions by combining the results from each component.

Therefore, VMD decomposition is an effective and necessary processing method to overcome the complexity of photovoltaic power data and improve its predictability.

Figure 17 and 21 show the comparison between the prediction results of VMD-CNN-LSTM (unoptimized) and VMD-KOA-CNN-LSTM models and the actual power curve. The unoptimized VMD-CNN-LSTM model has improved, can extract more features and respond more sensitively, but there are still details of deviation.

The significance and algorithm advantages of KOA optimization of CNN-LSTM hyperparameters

The significance of hyperparameter optimization: In photovoltaic power prediction, the CNN-LSTM model involves several key hyperparameters, including the number and size of convolutional kernels in the CNN layer, the number of hidden units and layers in the LSTM layer, dropout rates, and optimizer parameters such as learning rate and batch size. Traditional manual parameter tuning or grid search is inefficient in such a high-dimensional parameter space and can easily lead to local optima. Moreover, there is a strong coupling effect among these hyperparameters. For example, when the number of CNN convolutional kernels increases, the corresponding number of LSTM hidden units and learning rate need to be adjusted accordingly; otherwise, issues like gradient disappearance or overfitting may arise. Optimizing each parameter individually cannot capture this interdependence, whereas KOA's global search mechanism can consider the synergistic effects of all parameters simultaneously.

(2) The advantages of KOA over other optimization algorithms: KOA is based on the Keplerian laws of astrophysics, and its mechanism of 'planets' orbiting the 'optimal solution sun' has a clear physical basis. This avoids the randomness in parameter settings found in other heuristic algorithms. Parameters such as gravitational coefficients and orbital radii have clear physical explanations, making them easier to understand and adjust. Compared to other optimization algorithms, KOA converges faster and can achieve global optimization, leading to more robust parameter configurations.

The prediction curve of the research model is highly consistent with the actual situation, and the stable or fluctuating period is accurately predicted, which is almost coincident. It intuitively verifies its excellent performance in dealing with photovoltaic power problems, and provides guarantee for the stable operation and optimal scheduling of the power station.

**Reviewer#2, Concern # 1: Page 1 and further: the authors refer to the KOA as inspired by Kepler’s laws of planetary motion. Considering that the same source of inspiration is used by the Gravitational Search Algorithm (GSA), the authors should refer also to GSA in their review. A brief comparison between KOA and GSA should b added.**

**Author response:** Thank you very much for taking the time to review our article. We apologize for any errors we may have made. We have added a brief introduction to the gravitational search algorithm (GSA) and a concise comparison between KOA and GSA in the introduction, based on your feedback. The gravitational search algorithm (GSA), proposed by Iranian scholar Esmat Rashedi and colleagues in 2009, is a meta-heuristic optimization algorithm based on physical principles. Its inspiration primarily comes from Newton's law of universal gravitation and Newton's second law of motion, using the simulation of gravitational interactions and movement patterns in the universe to solve optimization problems. Compared to genetic algorithms (GA) and particle swarm optimization (PSO), the gravitational search algorithm (GSA) outperforms KOA in terms of convergence speed and global search capability, making it particularly suitable for optimizing high-dimensional parameter spaces. Please review it.

**Author action:**

**(Paragraph 3, first line of the introduction)**

**The gravitational search algorithm (GSA), proposed by Iranian scholar Esmat Rashedi and colleagues in 2009, is a meta-heuristic optimization algorithm based on physical principles. Inspired by Newton's law of universal gravitation and the second law of motion, GSA solves optimization problems by simulating the gravitational interactions and movement patterns of objects in the universe. Mohamed Abdel-Basset et al. published in the journal Knowledge-Based Systems in May 2023 that, inspired by Kepler's laws of planetary motion, they simulated the orbital motion of planets for global optimization. Compared with genetic algorithm (GA), particle swarm optimization (PSO) and gravity search algorithm (GSA), KOA performs better in convergence speed and global search ability, and is especially suitable for the optimization of high-dimensional parameter Spaces.**

**Reviewer#2, Concern # 2 :Pages 2 and 3, subsection 2.1: in Step 1, the authors refer ti i and N as „planets”, while further they refer the same parameters as being „objects”. Moreover, further away, in Step 3, they use the term „target planet”. Please, use a unique term.**

**Author response:** Thank you very much for taking time out of your busy schedule to review our article. We are sorry that we made some mistakes. We have changed it to "planet" according to your opinion to keep the context consistent and avoid misunderstanding by readers. Please review it.

**Author action:**

**(sect. 2.1, 10th line)**

**The orbital eccentricity (e) of each i-th planet is initialized using the equation**

**(Section 2.1, line 13)**

**Finally, initialize the orbital period T of each i-th planet.**

**Reviewer#2, Concern # 3 :Page 3, eq. (3) as compared to other equations: explain the difference between parameter „r” and „rand[0,1]”.**

**Author response:** Thank you very much for taking the time to review our article amidst your busy schedule. We apologize for any errors we may have made. We have processed equations (2) and (3) separately based on your suggestions. In these equations, the parameters 'r' and 'rand[0,1]' are not the same. rand_{[0,1]} is a uniform distribution random number generator that specifically generates random decimals within the [0,1] range. In equation (2), it is used to initialize e_i, which introduces basic random disturbances to the model, with values strictly limited between 0 and 1. r is a normally distributed random number (as clearly stated in the text, 'generated according to the normal distribution'). In equation (3), |r| is used to define the orbital period T_i. The key feature of the normal distribution is its symmetrical distribution around the mean, theoretically extending to positive and negative infinity (though it is truncated based on the model's requirements). This is fundamentally different from the uniform distribution logic of rand_{[0,1]}. Please review this.

**Author action:**

**(sect. 2.1, 10th line)**

**The expression is as follows:**

**ei=rand[0,1],i=1,...,N	(2)**

**In the formula, rand_([0,1]) is used to generate random numbers from 0 to 1.**

**Finally, initialize the orbital period T of each i-th planet. The expression is as follows:**

**Ti=|r|,i=1,...,N	(3)**

**In the formula, r is a number randomly generated based on the normal distribution.**

**Reviewer#2, Concern # 4 :Page 3, text after eq. (4):chek notation M_S.**

**Author response:** Thank you very much for taking time out of your busy schedule to review our article. We are sorry that we have made some mistakes. We have checked the symbols according to your opinion and changed the symbols to correct symbols $\overline{M}_{s}$. Please review it.

**Author action:**

**(sect. 2.1, line 19)**

In the formula, and represent the normalized values of Ms and mi, representing the mass of the sun Xs and the mass of the planet Xi respectively;

**Reviewer#2, Concern # 5 :Page 3: explain the rational of formula in eq. (6).**

**Author response:** Thank you very much for taking the time to review our article amidst your busy schedule. We apologize for any errors we may have made. We have reviewed formula (6) based on your feedback and added the missing symbols and numbers. Formula (6) is essentially the 'Velocity/Position Update Model' of the Kepler optimization algorithm, which is reasonable in several ways: it adapts the optimization process by using a segmented logic to cover the 'exploration-exploitation' dual phase, meeting the core requirements of intelligent optimization algorithms; it simulates celestial motion by using symbolic mapping and segmented calculations to mimic the 'diffusion-convergence' characteristics of planetary orbits; it ensures mathematical consistency through bias compensation (to simulate gravity) and control adjustment (to simulate orbital parameters), thereby establishing optimization rules that resemble celestial motion. Please review it.

**Author action:**

**(Section 2.1 Formula (6))**

**Reviewer#2, Concern # 6 :Page 3, eq. (6) and text after it: do not use small and capital letters for the same variable/parameter: see v cs. V, x vs. X etc**

**Author response**:Thank you very much for taking time out of your busy schedule to review our article. We are sorry that we made some mistakes. We have modified the case of equation (6) and the following text according to your opinion, and used the same case for the same variable or parameter. Please review it.

**Author action:**

**(Section 2.1 Formula (6))**

In the formula, 𝑉𝑖(𝑡) represents the velocity of the object Xi at time t; r3 and r4 are random numbers ranging from 0 to 1, and is a random value vector containing values between 0 and 1. and represent the solutions randomly selected from the population; μ(t) represents the gravitational constant;ε is a small value that prevents the error of division by zero; ai is the semi-major axis of the elliptical orbit of object i at time t.Ri (t) represents the distance between the Sun Xs and the target planet Xi at time t; Ri−norm(t) represents the normalized value of the Euclidean distance between the Sun Xs and the target planet Xi at time t.
